# Non-Synonymous and Synonymous Coding SNPs Show Similar Likelihood and Effect Size of Human Disease Association

**DOI:** 10.1371/journal.pone.0013574

**Published:** 2010-10-22

**Authors:** Rong Chen, Eugene V. Davydov, Marina Sirota, Atul J. Butte

**Affiliations:** 1 Department of Pediatrics, Stanford University School of Medicine, Stanford, California, United States of America; 2 Lucile Packard Children's Hospital, Palo Alto, California, United States of America; 3 Department of Computer Science, Stanford University School of Medicine, Stanford, California, United States of America; East Carolina University, United States of America

## Abstract

Many DNA variants have been identified on more than 300 diseases and traits using Genome-Wide Association Studies (GWASs). Some have been validated using deep sequencing, but many fewer have been validated functionally, primarily focused on non-synonymous coding SNPs (nsSNPs). It is an open question whether synonymous coding SNPs (sSNPs) and other non-coding SNPs can lead to as high odds ratios as nsSNPs. We conducted a broad survey across 21,429 disease-SNP associations curated from 2,113 publications studying human genetic association, and found that nsSNPs and sSNPs shared similar likelihood and effect size for disease association. The enrichment of disease-associated SNPs around the 80^th^ base in the first introns might provide an effective way to prioritize intronic SNPs for functional studies. We further found that the likelihood of disease association was positively associated with the effect size across different types of SNPs, and SNPs in the 3′untranslated regions, such as the microRNA binding sites, might be under-investigated. Our results suggest that sSNPs are just as likely to be involved in disease mechanisms, so we recommend that sSNPs discovered from GWAS should also be examined with functional studies.

## Introduction

Thousands of DNA variants have been identified on more than 300 diseases and traits in over 500 genome-wide association studies (GWASs) in the past five years. Some of them have been validated using high-quality deep sequencing, but many fewer of them have been validated functionally. DNA variants that were pursued for functional validation are usually those that were predicted to lead to significant amino acid changes, such as non-synonymous coding Single Nucleotide Polymorphism (nsSNPs). It is an open question whether synonymous coding SNPs (sSNPs) and other non-coding SNPs can lead to as high odds ratio as nsSNPs.

To answer the question of relative significance of sSNPs and nsSNPs, we need a catalog of known disease SNPs. There are many current gene-based resources containing knowledge on DNA variants associated with human diseases, such as The Human Gene Mutation Database (HGMD) [1], Genetic Association Database (GAD) [2], and Online Mendelian Inheritance in Man (OMIM) [3]. However, most of these resources, such as GAD and OMIM, do not report specific base-pair coordinates or dbSNP IDs. As of this writing, the professional version of HGMD only reports 1631 dbSNP IDs, which are embedded in free text comments, and do not report other essential information, such as p-value, odds ratio, and sample size.

Recently, the National Heart, Lung and Blood Institute (NHLBI) [4] and National Human Genome Research Institute (NHGRI) [5] released GWAS catalogs that were build from curated information from the published literature reporting thousands of DNA variants for human diseases. However, the NHLBI catalog only reports the p-values for the associations, and does not report the effect sizes, as indicated by the odds ratios. The NHGRI catalog does report both the p-value and the odds ratio, but does not report the context of that odds ratio, or specifically the two genotypes or alleles being compared. We argue that both the likelihood and the effect size need to be evaluated, and suggest that the effect size is going to be more comparable across studies, due to its independence from the sample size. In addition, both of these online catalogs ignore candidate gene studies. A recent report[6] showed that genetic associations proposed in the candidate gene era and replicated in GWASs had significantly larger effect size than those newly discovered in the GWASs, suggesting that the genetic associations identified from candidate studies should not be discarded. Furthermore, many candidate gene studies were performed in the post GWAS era, by deep sequencing around GWAS loci. Therefore, we found no comprehensive resource of human disease-associated DNA variants covering both GWASs and candidate studies, prior to this study.

We built a quantitative human disease-SNP association database, curating from 2,113 publications. In this study, we surveyed across the database and compared the likelihood and effect size of human disease-SNP association among nine types of SNPs, including nonsense, nsSNPs, sSNPs, and SNPs in the 5′-untranslated region (UTR), 3′-UTR, near 5′, near 3′, intronic, and intergenic regions.

## Results

**Figure 1:**
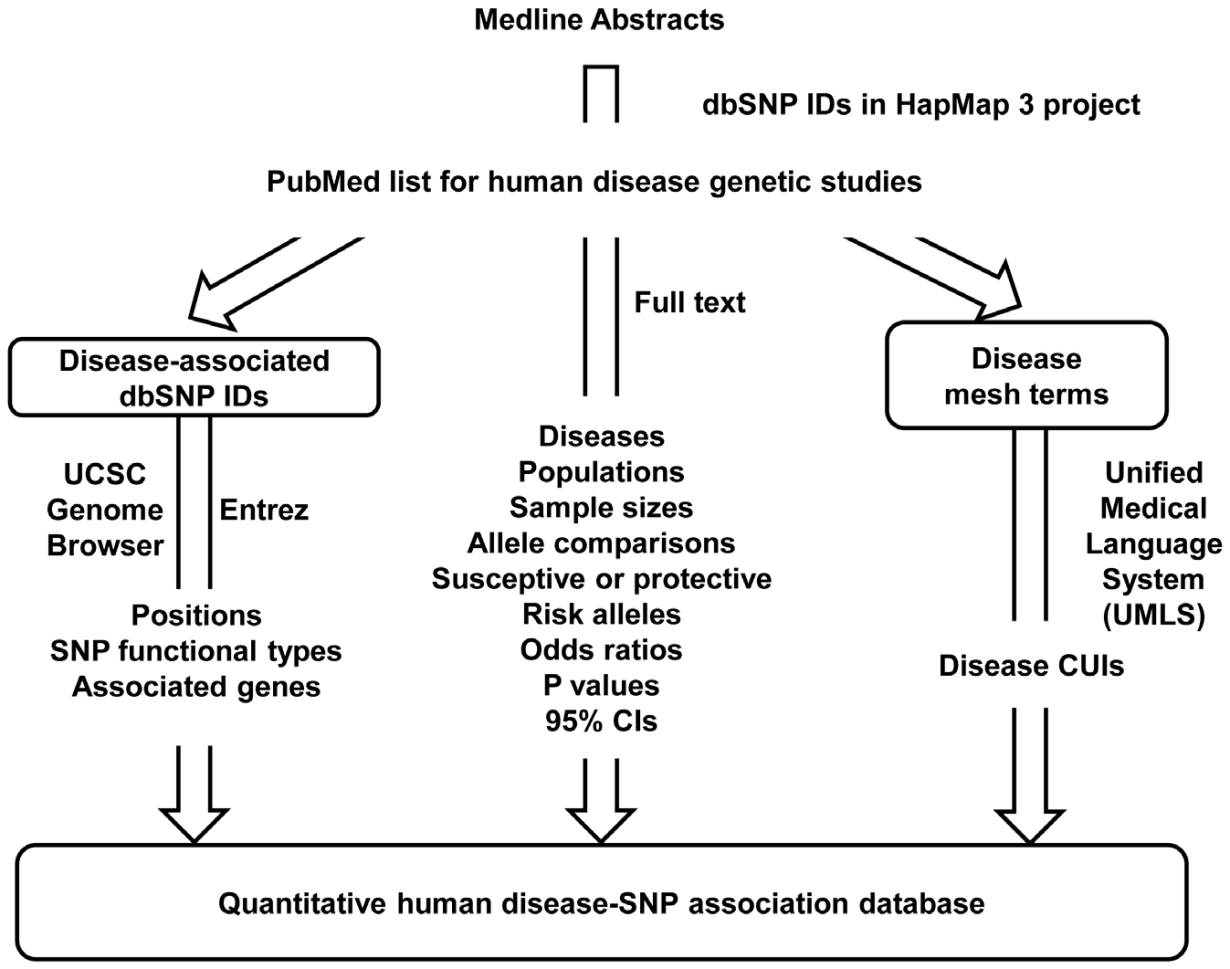
A curated quantitative disease-SNP association database. Starting from a list of all SNPs measured in the HapMap 3 project, we searched for their presence in all Medline abstracts, eliminating non-human studies. Significant SNP-disease associations were manually curated from the full text, and reviewed four rounds. SNP IDs were annotated using the UCSC genome browser for positions and function types and annotated using Entrez for associated genes. Disease mesh terms were compared with the Unified Medical Language System (UMLS) to select concept unique identifiers (CUIs).

As previously described [7], starting from a list of Medline abstracts that contains a dbSNP ID measured in the HapMap 3 projects[8], we manually curated 2,113 publications, and recorded more than 100 features of the disease-SNP associations, including the disease name (e.g. coronary artery disease), specific phenotype (e.g. acute coronary syndrome in coronary artery disease), study population(e.g. Portuguese), case and control population (Coronary artery disease patients vs. healthy patients), genotyping technology, major/minor alleles, odds ratio, 95% confidence interval of the odds ratio, published p-value, and genetic model (**Fig. 1**). By categorizing studies based on similar diseases, we manually extracted the disease Medical Subject Heading (MESH) terms [9], and mapped them to the Concept Unique Identifiers (CUI) in the Unified Medical Language System (ULMS) [10] to standardize disease names. We then annotated all SNPs using the UCSC Genome Browser and NCBI Entrez to retrieve the chromosome locations, functional types, and associated genes for each.

As of this writing, we have cataloged 21,429 statistically significant human disease-SNP associations from 2,113 publications. From this set, we found 45% of these variants had odds ratios described in their publications (**Table 1**). Five thousand, two hundred and seventy SNPs measured in the HapMap 3 project[8] were significantly associated with 375 different diseases, which accounts for 0.13% SNPs and 7.28% genes measured in the HapMap 3 project. Type 2 diabetes is the most studied diseases, associated with 514 SNPs across 397 studies.

**Table 1:** Statistics of the curated quantitative human disease-SNP association database.

	Associations	PubMed	Diseases	SNPs	Genes	Percentage of genes in HapMap3
All	21,429	2,113	375	5,270	1,588	7.28%
With odds ratio	9,574	1,255	292	2,764	1,003	4.60%

**Figure 2:**
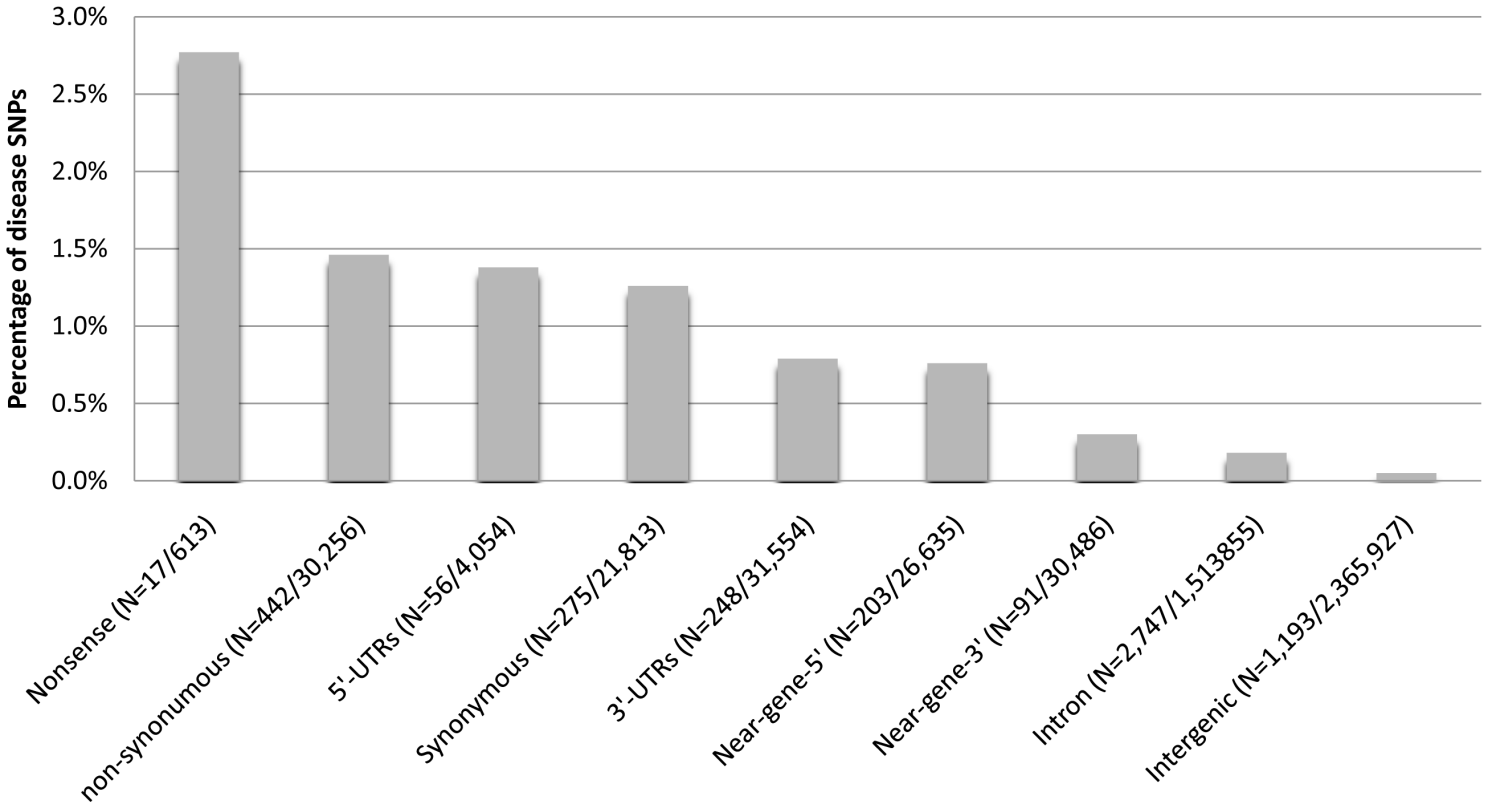
Impact of SNP types on the likelihood of disease association. For each of the nine SNP types, the likelihood of disease associations was estimated as the percentage of disease-associated SNPs among all SNPs measured in the HapMap 3 project. Similar likelihoods were observed among nsSNPs, sSNPs, and SNPs in the 5′-UTR. The number of disease-associated SNPs and SNPs measured in the HapMap 3 project were specified in the parenthesis.

We evaluated the impact of SNP types on disease association using the likelihood and effect size. We estimated the likelihood of disease association for each SNP type using the percentage of disease-associated SNPs known in the literature on two different references. First, we calculated the likelihood of being associated with human disease for six types of SNPs from the HapMap 3 project (**Fig. 2**). As expected, nonsense variants, which cause a premature stop, were most likely to be associated with diseases with 2.77% probability. Interestingly, 1.46% of nsSNPs, 1.38% of SNPs within the 5′-UTR region, and 1.26% of sSNPs (1.26%) have also been known to associate with human disease. The result indicates that sSNPs and SNPs in the 5′-UTR regions were as likely to be associated with disease as nsSNPs. Out of 5,272 HapMap SNPs that were statistically significantly associated with disease, only 8.7% of them are nonsense or nsSNPs, causing amino acid changes. If investigators attempt validation and functional studies on amino-acid changing variants only, we predict they will likely miss more than 90% disease-associated variants.

Second, we calculated the likelihood of disease association for each SNP type on six current human genotyping platforms (**Table 2**). Similar with the results using the SNPs measured in the HapMap 3 project, 2.28–16.67% of nonsense SNPs had been discovered to associate with disease, which is significantly higher than all other SNP types. Combing SNPs measured in all six platforms, we found 2.10% of nonsense SNPs, 1.57% of nsSNPs, 1.35% of SNPs within 5′-UTR, and 1.16% of sSNPs had been reported to associate with human disease. Therefore, nsSNPs and sSNPs shared similar likelihood of being associated with human disease on current genotyping platforms.

**Table 2:** Likelihood of disease association for SNPs on current genotyping platforms.

Platform	Nonsense	nsSNP	sSNP	5′-UTR	3′-UTR	Near-gene-5′	Near-gene_3′	Intronic	Intergenic
Affy 6.0	5.56%	2.51%	1.80%	2.15%	0.94%	0.63%	0.86%	0.27%	0.07%
Affy 5.0	16.67%	3.33%	2.29%	0.82%	0.84%	0.65%	1.09%	0.32%	0.10%
Affy 500k	16.67%	3.15%	2.19%	0.74%	0.76%	0.66%	1.02%	0.30%	0.10%
Illumina Omni1	3.05%	2.00%	1.31%	1.54%	1.01%	0.79%	1.29%	0.39%	0.12%
Illumina 1M	2.28%	1.85%	1.42%	1.56%	0.94%	0.89%	1.10%	0.38%	0.12%
Illumina 660W	3.12%	3.88%	2.37%	2.13%	1.74%	1.32%	1.56%	0.45%	0.16%
Combined array*	2.10%	1.57%	1.16%	1.35%	0.84%	0.71%	0.96%	0.30%	0.08%

*A hypothetical platform combined from the above six platforms to report the likelihood of SNPs being associate with human disease for each SNP type.

**Figure 3:**
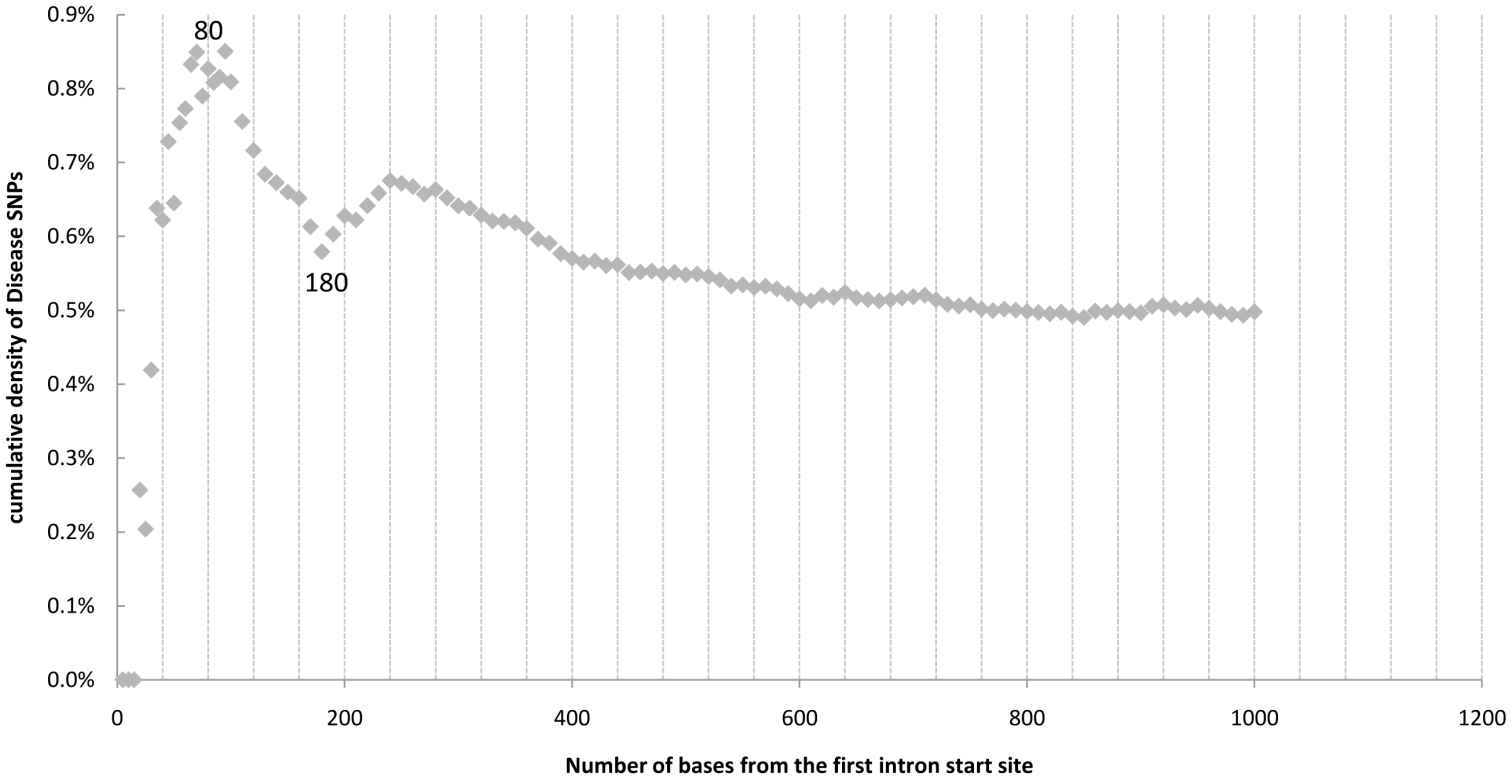
Cumulative density of disease-associated SNPs in the first intron. The cumulative densities of disease-associated SNPs were plotted against the distance from the first intron start site. The density was peaked at the 80^th^ base from the first intron start site.

There were still 2,747 or 52% disease-associated SNPs within the intronic regions; therefore, prioritizing disease SNPs in the intronic region would be essential for the identification of novel disease variants. Using gene annotations from the UCSC genome browser we identified SNPs located within the first intron of a gene, and their position relative to the start of the intron. We analyzed all 1,497,869 HapMap intronic SNPs with all experimentally validated transcripts and 31% of them fell into the first intron. SNPs in the first intron were 1.2 times more likely to be associated with disease than SNPs in the non-first introns (p = 2.2×10^−8^, hypergeometric test). We then plotted the cumulative density of disease SNPs along the first 1000 bases of the first intron. The likelihood of disease association was dramatically increased to a peak of 0.85% at the 80th bases, quickly decreased back to 0.58% at the 180^th^ base, and gradually stabilized at 0.5% (**Fig. 3**). Investigation around the 80^th^ base in the first introns might provide an effective way to prioritize intronic SNPs for functional studies.

**Figure 4:**
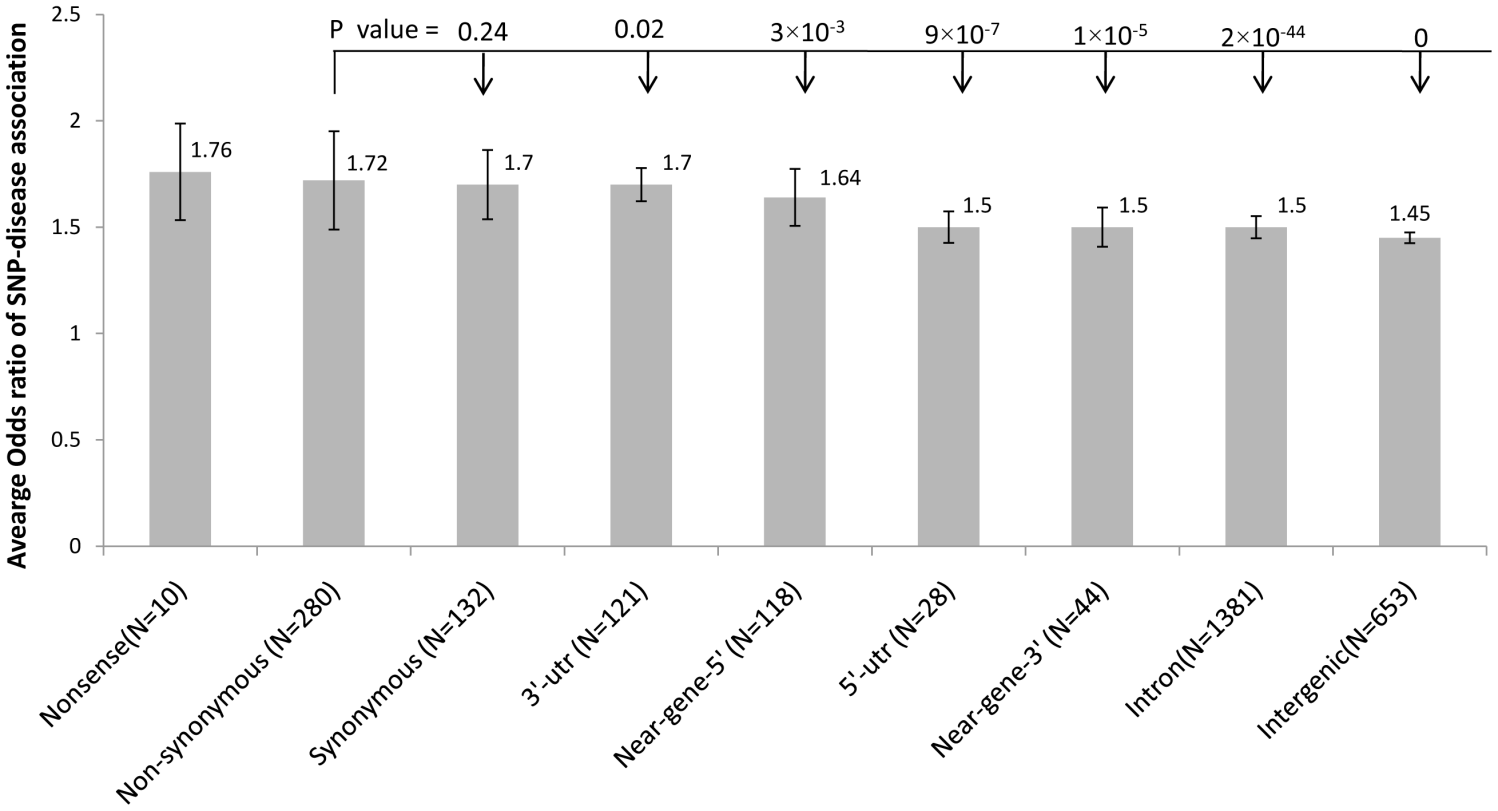
Impact of SNP types on the effect size of SNP-disease association. The median odds ratio±standard error was calculated for each of the nine SNP types using 9,574 curated SNP-disease associations. The number of distinct SNPs was specified in the parenthesis. The odds ratios of nonsense SNPs were significantly higher than those of other SNP types (p<0.05, Mann-whiney U test). The p-values between the odds ratios of nsSNPs and other type of SNPs were shown in the figure.

We then evaluated the effect size of disease association among nine SNP types by comparing the odds ratios of 9,574 disease-SNP associations. As expected, nonsense SNPs had a median odds ratio of 1.76±0.227, significantly higher than those of all other SNP types (p<0.05, Mann-Whitney U test, **Fig. 4**). Surprisingly, nsSNPs shared a similar median odds ratio with sSNPs (1.72 vs. 1.70, p = 0.24, Mann-Whitney U test), and had a significantly higher median odds ratio than the remaining six SNP types, with p-values ranging from 0.02 to 2×10^-44^. Therefore, nsSNPs and sSNPs not only shared similar likelihood of disease association, but also shared very similar effect size of disease association.

**Figure 5:**
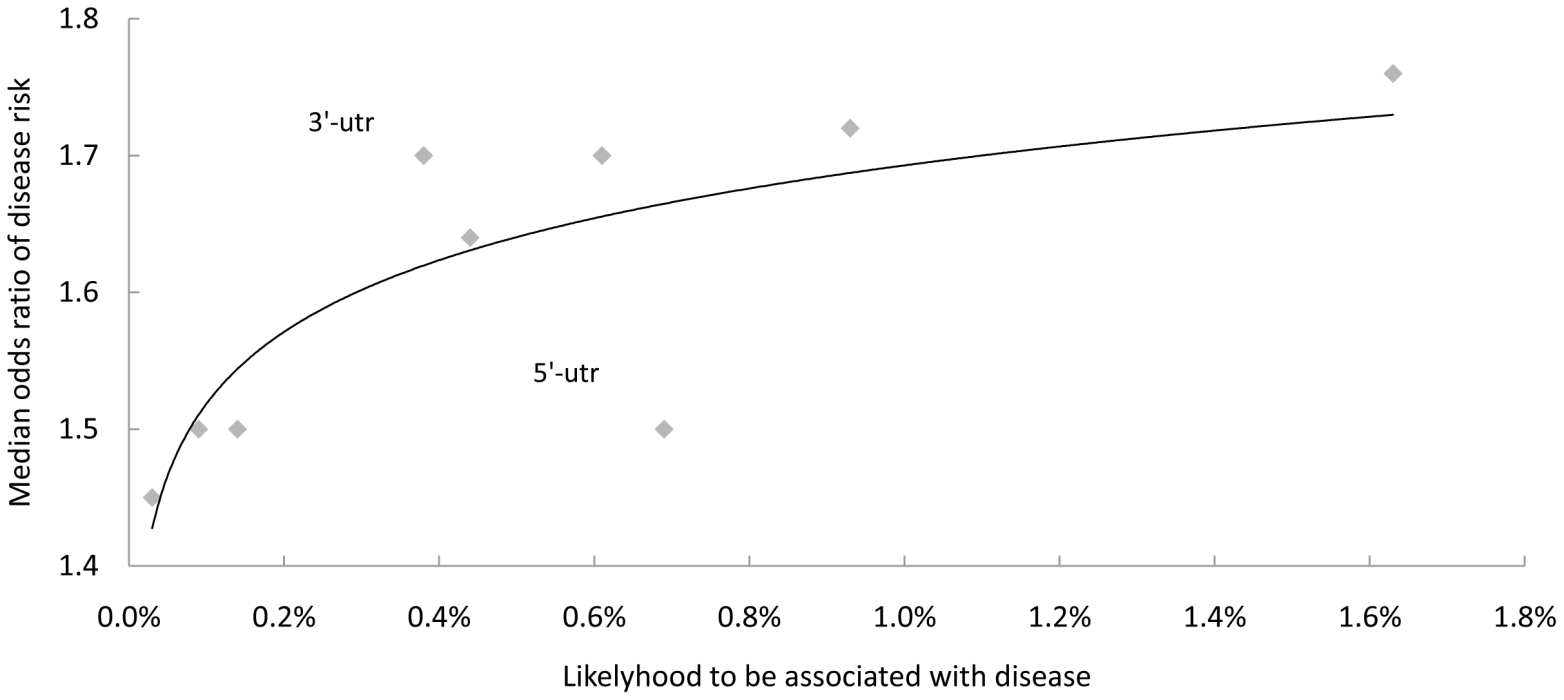
SNPs in the 3′-UTR were under-investigated. The median odds ratio of SNP-disease associations was plotted against the percentage of disease-associated SNPs for each of the nine SNP types. Assuming the median odds ratio will not be increased with more studies performed, the percentage of disease-associated SNPs will likely be increased to fit the curve.

Finally, we plotted the likelihood against the median odds ratio for all nine SNP types (**Fig. 5**). Interestingly, the effect size and likelihood of disease associations fit almost perfectly along the curve. The stronger diseases association a SNP type had, the more likely it was to be associated with disease. The only outliers were SNPs in the UTR regions. If we assumed that the association effect size or odds ratios would not be changed when more studies were performed, 3′-UTR was clearly under-investigated for disease association and 5′-UTR was over-investigated.

## Discussion

We conducted a broad survey across 21,429 human SNP-disease associations curated from 2,113 publications, and compared the likelihood and effect size of disease association among nine types of SNPs. We found that sSNPs shared similar likelihood and effect size of human disease association compared to nsSNPs, using SNPs measured in the HapMap 3 project and six current genotyping platforms as the reference.

We acknowledge that these sSNPs might not be causal, but we also found that they are not likely to be in linkage disequilibrium (LD) with another undiscovered causal nsSNPs due to three reasons. First, we curated the 21,429 SNP-disease associations from the full text, tables, and abstracts in the publications, not from the raw genotyping data. We suspect investigators would have preferred and reported nsSNPs than sSNPs if the nsSNPs had reached a threshold of statistical significance. Second, a recent study suggested that uncommon or rare genetic variants can easily create synthetic associations that are credited to common variants[11]. If most reported sSNPs were synthetic from causal nsSNPs, their effect size would be lower than that of nsSNPs, which was different from our observation here. Furthermore, many sSNPs had been consistently found to associate with human disease in multiple different ethnicities, indicating that they were not likely to be synthetic. For example, rs3810936, a synonymous SNP in *TNFSF15* was found to be significantly associated with Crohn's disease in Japanese[12], [13], Korean[14], and Caucasian [15]. Recently, several studies also suggested that synthetic associations do not underlie many reported GWAS associations[16], [17]. Finally, sSNPs might be causal through influencing promoter activity and the conformation and stability of pre-mRNAs[18], or changing the rate of protein folding[19]. A recent review summarized the experimental evidence and mechanism on how sSNPs altered the structure, function, and expression levels of proteins[20]. Therefore, we believe that sSNPs are highly likely to be involved in the disease mechanisms, and should be investigated in the functional studies after GWASs.

We also found that the effect size of disease association was positively associated with the likelihood of disease association for different SNP types. This is reasonable because the larger the effect size that a SNP has, the more likely it would have been discovered with genome-wide significance. Interestingly, only limited number of SNPs in the 3′-UTR regions were discovered for disease association in comparison with their relatively large effect sizes, suggesting 3′-UTR might be under-investigated. A recent study analyzed the genome-wide gene expression on 270 HapMap individuals and found that SNPs in the UTR regions showed consistently increased population heterozygosity and were linked with disease susceptibility[21]. Further investigation on SNPs in the 3′-UTR region, especially the microRNA binding sites[22], will likely identify many novel variants for missing heritability.

## Materials and Methods

### Curating human disease-associated DNA variants

As previously described[7], staring from a list of all SNPs measured in the HapMap 3 project [8], we searched for the presence of these rsIDs in all Medline abstracts, examined the full text of these publications, and manually extracted SNPs that were reported as statistically significantly associated with human disease. We curated all SNPs described in the papers, but only SNPs that were measured in the HapMap 3 project were used in this paper [8]. For SNPs whose dbSNP IDs, or rsIDs were not reported in the publications, they were manually compared with the sequences in the NCBI Human dbSNP build 130 [23] to ensure a valid dbSNP ID for every SNP in our database.

### Categorizing studies on similar diseases

To enable the integration of multiple studies on similar diseases, we mapped the disease/phenotype names in our association database to the Unified Medical Language System (UMLS) Concept Unique Identifiers (CUIs) [10]. To ensure the difficult process of matching disease names to UMLS CUI was performed with high quality, we manually selected the best descriptive disease names from the Medical Subject Heading (MeSH) terms [9] associated with each paper during the curation, and manually examined the matching UMLS CUIs. Gradually, we built up a standardized list of commonly studied disease names, which smoothed the automated post-processing.

### Annotating and categorizing SNPs

All SNPs measured in the HapMap 3 project were annotated using the UCSC genome browser [24] with the dbSNP build 130 to retrieve their physical locations and functional types. SNPs that were located within or near a gene were annotated using the NCBI Entrez. We parsed the “Known Genes” track of the UCSC genome browser[24] and extracted intron annotations from each gene, noting the index of each intron in the direction of transcription. All intronic SNPs were mapped to transcripts and labeled according to whether they fell inside the first intron and by distance from the intron start site.

### Calculating likelihood and effect size of disease association

We estimated the likelihood of disease association as the percentage of SNPs being associated with human disease among all SNPs measured in the HapMap 3 project or six genotyping platforms, including Affymetrix Human GenomeWide SNP arrays (6.0, 5.0, & 500K), and Illumina Human Omni1-Quad1, 1M-Duov3, and 660W-Quadv1. All annotation files were downloaded from Affymetrix and Illumina company web sites. The effect size of disease association was estimated as the median odds ratio among all curated SNP-disease associations for each SNP type.
